# The Role of Extracellular Vesicles in Melanoma Progression

**DOI:** 10.3390/cancers14133086

**Published:** 2022-06-23

**Authors:** Evelyn Lattmann, Mitchell P. Levesque

**Affiliations:** Department of Dermatology, University of Zurich, University Hospital Zurich, Wagistrasse 18, CH-8952 Schlieren, Switzerland; mitchell.levesque@usz.ch

**Keywords:** exosomes, extracellular vesicles, EV, microvesicles, melanosome, melanoma, metastasis, miRNA, biomarker

## Abstract

**Simple Summary:**

Melanoma is produced by the malignant transformation of the pigmented cells in the skin. It is the deadliest form of skin cancer and a global medical burden. Recent research suggests that extracellular vesicles improve diagnosis and treatment of melanoma. Extracellular vesicles are virus-like vehicles that are released into the blood and other body fluids by most cell types, including cancer cells. They sequester molecular substances from the cytoplasm and transport them as messengers to target cells. Thanks to these properties, extracellular vesicles provide a molecular fingerprint of the cell of origin and can serve as biomarkers for cancer diagnosis or prognosis. In addition, molecular signals exchanged through extracellular vesicles between cancer cells and the tumor environment can reveal signaling pathways that are important for cancer progression. In this review we give a general overview of extracellular vesicles and focus on their impact on melanoma progression and potential use as biomarkers for monitoring and treating melanoma.

**Abstract:**

Cutaneous melanoma arises from a malignant transformation of the melanocytes in the skin. It is the deadliest form of skin cancer owing to its potential to metastasize. While recent advances in immuno-oncology have been successful in melanoma treatment, not all the patients respond to the treatment equally, thus individual pre-screening and personalized combination therapies are essential to stratify and monitor patients. Extracellular vesicles (EVs) have emerged as promising biomarker candidates to tackle these challenges. EVs are ~50–1000-nm-sized, lipid bilayer-enclosed spheres, which are secreted by almost all cell types, including cancer cells. Their cargo, such as nucleic acids, proteins, lipids, amino acids, and metabolites, can be transferred to target cells. Thanks to these properties, EVs can both provide a multiplexed molecular fingerprint of the cell of origin and thus serve as potential biomarkers, or reveal pathways important for cancer progression that can be targeted pharmaceutically. In this review we give a general overview of EVs and focus on their impact on melanoma progression. In particular, we shed light on the role of EVs in shaping the tumor–stroma interactions that facilitate metastasis and summarize the latest findings on molecular profiling of EV-derived miRNAs and proteins that can serve as potential biomarkers for melanoma progression.

## 1. Introduction

Malignant melanoma is the most fatal form of skin cancer and arises from malignant transformation of the melanocytes, which are the pigment-producing cells in the skin. In most cases it is induced by UV irradiation and thus characterized by a high tumor mutational burden. The most frequent mutations reside in genes regulating the MAPK pathway; BRAF accounts for approximately 50% of all tumor mutations and NRAS for around 15% [1,2]. The most common mutation leads to a V600E substitution and is found in approximately 80% of BRAF-mutated patients [3]. Other frequent genetic alterations associated with melanoma include the CDK4/CDKN2A axis, inactivation of p53 and NF1, as well as SF3B1 and TERT promoter mutations [4]. Due to the importance of the MAPK pathway in melanoma, the first targeted therapies were small-molecule inhibitors of BRAF (vemurafenib, dabrafenib), which were followed by combination therapies with BRAF and MEK inhibitors [5]. However, several melanomas appeared intrinsically resistant or developed resistance within a few months of treatment initiation. Current therapy has predominantly shifted to immune checkpoint blockers (ipilimumab, nivolumab, pembrolizumab), which release the natural brakes on the immune system and allow CD8+ T cells to recognize and eliminate melanoma cells [6,7,8]. Despite a substantial genetic contribution, epigenetic heterogeneity and plasticity are crucial hallmarks associated with melanoma progression and resistance formation. In particular, changes in miRNAs have been associated with melanoma [9]. miRNAs are small ~21–24-nucleotide-sized non-coding RNAs that regulate gene expression by either inhibiting mRNA translation [10,11,12,13] or by causing RNA degradation [14,15,16], generally by binding to the 3′ untranslated region (3′ UTR) of mRNAs. In 1993, Lee and colleagues discovered the first miRNAs in *C. elegans* and since then more than 2000 human miRNAs have been identified [11]. Due to promiscuous binding of miRNAs to mRNA target sequences, miRNA-mediated expression changes can impact hundreds of genes [17]. Thus, unique patterns of altered miRNAs can provide molecular fingerprints of cancers [18]. However, besides a cell-autonomous role in the tumor, miRNAs also act in a paracrine fashion to regulate interactions with the tumor microenvironment. Importantly, to protect miRNAs from degradation in the extracellular space they are bound to (lipo-) proteins and nanoparticles or are enclosed in extracellular vesicles (EVs). In a seminal paper, Valadi and colleagues showed that miRNAs encapsulated by EVs can be taken up by target cells and induce gene expression changes in these cells [19]. This finding sparked several studies on EVs through which EVs emerged as important mediators of (cancer) cell communication. To date, EVs have been shown to shuttle a diverse set of molecules including proteins, RNA, DNA, lipids, and metabolites among cells. Furthermore, transport of whole organelles has been observed, as for example the transfer of mitochondria to neurons by extracellular astrocyte-derived particles [20] (reviewed in [21]). Besides the variability at the level of EV cargo, abundant heterogeneity of different vesicle types has been observed. This combination enables a vast complexity of cell-to-cell interactions. Accordingly, EVs appear to influence a plethora of cancerous processes ranging from the regulation of angiogenesis and fibroblast activation, to establishing organotrophic metastasis [22]. Moreover, several studies have identified functions of tumor-derived EVs in modulating interactions with the immune system. In melanoma, PDL-1 and FasL were expressed on tumor-derived EVs to suppress the antitumor effect of CD8+ T cells or induce Fas-dependent apoptosis of T cells, respectively [23,24]. A recent publication also showed that tumor-derived EVs can help establish pre-metastatic niches in lymph nodes through the activity of NGFR [25]. In this review we give an overview of the different types of EVs and summarize some of the key studies that have investigated the role of EVs in preparing the pre-metastatic niche. In the last section we focus on EV-derived cargo as conceivable biomarkers for translational oncology and potential applications to clinical practice.

## 2. Extracellular Vesicle Diversity

**Figure 1:**
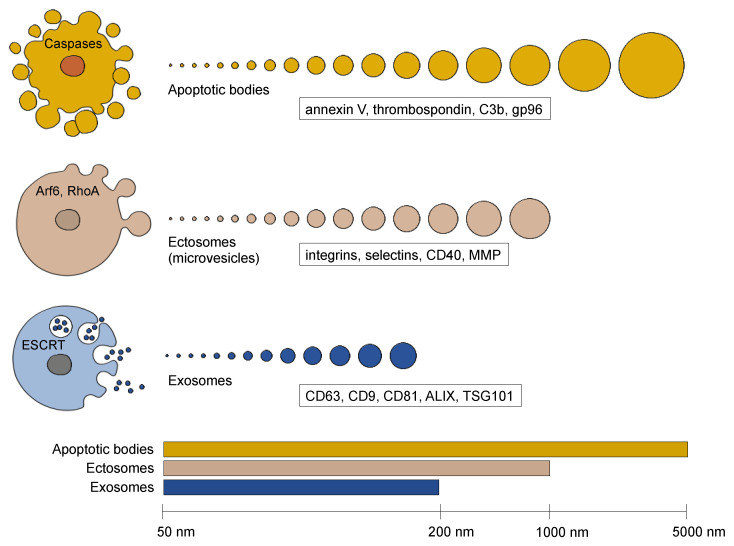
Extracellular vesicle diversity. Traditionally, EVs are classified into three major categories consisting of apoptotic bodies, ectosomes (microvesicles) and exosomes. Proteins indicated in rectangular boxes are referring to commonly used markers for the corresponding EVs.

EVs are lipid-bilayer structures that contain diverse cargo such as mRNAs, miRNAs, long non-coding RNAs, mitochondrial DNAs, single-stranded DNAs, double-stranded DNAs, proteins, metabolites, and lipids. Cargo can either be degraded or recycled to regulate cellular homeostasis or transferred from donor to recipient cells to facilitate cell–cell communication [19,26]. Similar to viruses, EVs are coated by surface proteins that are EV-specific and enable docking to recipient cells through ligand–receptor interactions. In the current literature, EVs are generally classified into three major groups (i.e., ectosomes, exosomes, and apoptotic bodies) based on their biogenesis, release pathways, size, content, and function (Figure 1) [27].

### 2.1. Apoptotic Bodies

Apoptotic bodies are EVs that are exclusively formed by dying cells in contrast to exosomes and ectosomes that are generated by living cells [28]. At the final phase of apoptosis, the cell divides into a variable number of apoptotic bodies that differ in size ranging from 50 nm up to 5000 nm in diameter [29]. The cargo of an individual apoptotic body consists of the cellular components that happened to be in the cytoplasmic protrusion that created it [30]. Therefore, some apoptotic bodies are almost entirely made of condensed nuclear chromatin, whereas others carry only cytoplasmic components. On their surface, apoptotic bodies display so-called “eat-me” signals which promote recognition and clearance by phagocytes. The functional role of apoptotic bodies in paracrine signaling remains to be investigated. A few studies show a role for vesicles derived from apoptotic endothelial cells in stimulating proliferation and differentiation of endothelial progenitor cells [31] and an antiapoptotic phenotype in vascular smooth muscle cells [32]. Horizontal transfer of DNA by apoptotic bodies to neighboring cells has also been observed among healthy (e.g., endothelial cells, fibroblasts, and macrophages) and tumor cells [33,34,35].

### 2.2. Ectosomes (Microvesicles)

Ectosomes, also referred to as microvesicles (MVs), are formed by outward budding of the plasma membrane, which generates a heterogenous population of vesicles in the size range of approximately 50 nm to 1 µm in diameter. Several reports have shown that MVs are associated with tumor invasiveness. Thus, many studies focused on tumor cells to study their formation. The budding of MVs occurs at specialized membrane regions that are enriched in plasma-membrane components such as integrins, HLA molecules and cell surface-associated proteolytic enzymes (e.g., metalloproteinases) [36]. Vesicle shedding is dependent on calcium and requires regulation of the cytoskeleton [37]. Several GTPases have been shown to govern both bud formation and cargo recruitment [38]. In invasive melanoma cells, ARF6-dependent recruitment of ERK to the plasma membrane led to activation of the myosin light chain kinase and subsequent MV release. ARF6 recruited cargo included MHC class I, β1-integrin receptors, as well as VAMP3 [39]. Downstream of ARF, RHO signaling was shown to be critical for tumor MV shedding and the process was antagonized by Rac1 [40]. Under hypoxic conditions, the GTPase Rab22 appeared to be implicated in the selective recruitment of proteins in breast cancer cells [41].

### 2.3. Exosomes

Exosomes are approximately 50–200 nm in diameter and are distinguished from ectosomes by their biogenesis pathway. In contrast to ectosomes that are formed by outward budding of the plasma membrane, exosomes are formed in the endocytic pathway by the inward invagination of late endosomal membranes. During endocytosis, multivesicular bodies (MVB) are formed through fusion of smaller vesicles. The emerging MVBs invaginate their confining membranes to generate intraluminal vesicles (ILVs), the precursor vesicles of exosomes. During this inward budding, ESCRT proteins and RNA binding proteins allow for specific incorporation of proteins and RNAs into the lumen of the forming ILVs. Upon fusion of the MVB with the plasma membrane, the ILV are released as exosomes. ILV formation and cargo recruitment are regulated by the endosomal sorting complex required for transport (ESCRT) machinery, which are four protein complexes (ESCRT-0, -I, -II, and -III) and associated proteins including the ATPase Vps4 and ALIX [42,43]. ESCRT-0 binds and sequesters the ubiquitinated cargo proteins and ESCRT-I and ESCRT-II induce membrane invagination [44]. Finally, ILV scission is regulated by the ESCRT-III complex and Vps4 in an energy-consuming step [45]. Besides the ESCRT machinery, the exosomal protein ALIX, which is associated with the ESCRT-I protein TSG101, is involved in ILV budding and abscission, and recruits exosomal cargo via interaction with syndecan [46,47,48]. Tetraspanins also participate in exosome biogenesis and protein loading and tetraspanins CD9, CD63, CD81, CD82 are highly enriched in exosomes [49]. In addition, lipid membrane composition also influences exosome formation. In particular, ceramide and neutral sphingomyelinase, which converts sphingomyelin into ceramide, were reported to be critical for ILV formation [50]. Although exosomes and ectosomes are distinguished at the level of their biogenesis, the operational definition for exosomes has been based on the presence of protein markers (Figure 1). These proteins were also listed in a position statement by the International Society of Extracellular Vesicles (ISEV), which published the “Minimal information for studies of extracellular vesicles” (MISEV2014) guidelines for working with exosomes [51]. However, recent research suggests a more nuanced EV marker distribution. In addition, current standard exosome isolation methods (reviewed in [52]) such as size-exclusion-chromatography (SEC) and ultracentrifugation, which enrich particles based on size, lead to EV populations of heterogenous composition. Therefore, the updated MISEV2018 position statement suggests to categorize EVs either based on their (i) physical condition (e.g., small EVs vs. middle/large EVs), (ii) biochemical composition (e.g., CD63+/CD81+ EVs) or (iii) conditions or cell of origin (e.g., hypoxic EVs) as long as specific markers of subcellular origin cannot be reliably established within the experimental system [53]. To follow that standard, this review will refer to exosomes and small MVs collectively as small EVs (sEVs).

### 2.4. Different Subtypes of EVs and EV-Like Nanoparticles

**Figure 2:**
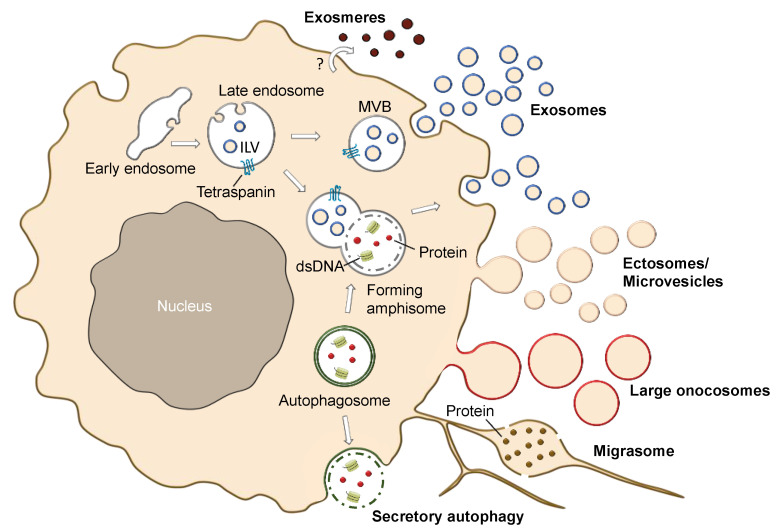
Example of EVs secreted by a melanoma cell. In addition to the traditional EV types (ectosomes, exosomes and apoptotic bodies), a more diverse set of EVs including exomeres, large oncosomes, migrasomes, and EVs derived from secretory autophagy are depicted. ILV refers to intraluminal vesicle; MVB annotates multivesicular bodies; the question mark implies that the biogenesis and secretion of exomeres are largely unexplored to date.

In addition to apoptotic bodies, ectosomes, and exosomes, there are several other types of vesicles and hybrids of fusion events (Figure 2). For example, when endocytic and autophagy pathways converge through fusion of endosomes and autophagosomes they form hybrids that are called amphisomes. Therefore, amphisomes display autophagy markers (e.g., LC3) as well as endosomal markers (e.g., Rab7, Rab5) [54]. Amphisomes can either fuse with lysosomes or secrete their cargo by membrane fusion [55,56]. However, autophagosomes can also be secreted without the formation of amphisomes, which is referred to as secretory autophagy. The role of secretory autophagy in melanoma has recently been reviewed [57]. Other particles that may contaminate exosome preparations are exomeres [58]. Exomeres are non-vesicular nanoparticles of up to 50 nm in size. They differ in protein, RNA, DNA and lipid content compared to exosomes [58,59]. The different profile might point to a distinct biogenesis pathway. However, their origin has not been explored yet. Furthermore, their role in melanoma still needs to be explored. Large oncosomes originate from non-apoptotic blebs and are large structures in the range of 1–10 µm in size [60]. They are formed upon cellular transformation by large protrusions of the plasma membrane. EGFR and AKT1 pathways have been shown to stimulate their formation [60]. Large oncosomes differ in their protein content from smaller EVs and show enrichment of enzymes involved in glucose, glutamine and amino acid metabolism [61]. In contrast to sEVs, these large EV structures were able to change glutamine metabolism upon transfer to recipient cells [57]. To our knowledge, oncosomes have not been studied in melanoma. However, given the link of large oncosomes to cell metabolism and EGFR/AKT1 pathways, their role in melanoma merits further exploration. Originally, large oncosome were simply referred to as “oncosomes”, but newer definitions of oncosomes label EVs, which carry transforming bioactive molecules such as oncogenic proteins [62]. Oncosomes which are typically smaller i.e., between 100–400 nm, derive their name from their “oncogenic” cargo. Other EVs defined by their functional origin include migrasomes (500 nm–3 microns), which are released from retracting fibers of migrating cells and are enriched in tetraspanin proteins [63,64]. NDST1, PIGK, CPQ, and EOGT were also shown to be putative markers of migrasomes [64].

In summary, there is a vast heterogeneity of EVs and EV-like structures that differ in their generation, marker expression, size, and subcellular origin. The combination of different parameters leads to an even larger complexity of EV populations and, therefore, we are only at the beginning of understanding the EV world. Moreover, vesicles can also be cell-type specific. Notably, melanocytes, which are the precursor cells of melanoma, contain EV-like structures, the so-called melanosomes, which are involved in translocation of pigments to the outer layer of the skin.

## 3. Melanosomes Are EV-like Organelles in the Skin

Melanocytes in the skin protect keratinocytes from UV radiation through the generation of a brown-black pigment eu-melanin that is transferred from the melanocytes to the keratinocytes where it locates around the sun-exposed side of the nuclei in a cap-like fashion to shield the keratinocyte DNA from UV-induced damage [65]. The transfer of eu-melanin to keratinocytes occurs through melanosomes, which are derived from endosomal membranes, despite their naming as lysosome-related organelles (LRO).

### 3.1. Melanosomes

Melanosome biogenesis occurs in four stages. Stage I consists of the formation of intraluminal vesicles and intraluminal proteinaceous fibrils. The main component of the fibrils is the premelanosome protein (PMEL) or silver locus protein homolog (SILV), also known as Pmel17/GP100, an integral membrane protein [66]. In stage II, the fibrils arrange in parallel sheet-like structures and the premelanosomes adopt an ellipsoidal shape. Melanin-synthesizing enzymes such as tyrosinase, tyrosinase-related protein-1 (TYRP1) and dopachrome tautomerase (DCT/TYRP2) are then transferred to the premelanosomes and newly synthesized melanin is deposited onto the fibrils (stage III). This thickening process continues until the internal structure is completely covered by melanin in the mature melanosome (stage IV) (reviewed in [67,68]). Several proteins of the endosomal machinery are involved at different stages of melanosome biogenesis. For example, CD63 is required for PMEL fibril formation which manifests on the surface of the ILVs of the early endosomes. Other proteins involved in melanosome generation include Rabs, SNARE, and BLOC complexes, the role of which have been reviewed in [68] and in [69], focusing on Rab proteins. Upon secretion of melanosomes, a process that is still not well understood, the transfer of the melanosomes from melanocytes to keratinocytes occurs along the cytoskeleton and involves microtubules and actin. The GTPase Rab27A, myosin Va and the linker protein melanophilin (also known as SLAC2a) have been shown to mediate the tethering of melanosomes to actin filaments [69]. Consequently, mutations in Rab27A, myosin Va and melanophilin can lead to impaired melanosome transport to keratinocyte and cause Griscelli syndrome, characterized by hypopigmentation of skin and hair [70]. To date, the uptake route to the keratinocyte remains controversial and four proposed models have recently been reviewed in [71].

### 3.2. The Role of Melanosomes in Melanoma Homeostasis and Inter-Cellular Communication

There is evidence that even after malignant transformation, melanoma cells can retain the function of melanosome biogenesis [72,73,74]. However, only a few studies address the functional relevance of melanosomes in melanoma. A role of melanosomes in melanoma cell homeostasis has been analyzed in the response to cytotoxic drug treatment. The study found that melanosomes can sequester cytotoxic drugs such as cis-diaminedichloroplatinum II (cisplatin) and increase melanosome-mediated drug export [75]. A function of paracrine signaling of melanosome has been reported too. In a seminal study, Dror and colleagues showed that melanosome cargo can be transferred to fibroblasts to stimulate tumor niche formation [76]. In that study, authors profiled melanosomes isolated from MNT-1 cells melanoma cells and found enrichment of several miRNAs. Five of the miRNAs (miR-149, miR-211, miR-23, miR-let7a, miR-let7b) that were enriched in mature compared to pre-mature melanosomes overlapped with melanoma-related miRNAs. Notably, miR-211, which showed the strongest enrichment, was further demonstrated to be transferred to fibroblast and induce a cancer-associated fibroblast (CAF) phenotype by activating pro-inflammatory pathways, migration and proliferation. A mechanism was proposed whereby miR-211 transferred by melanosomes to fibroblasts targets IGF2R in the recipient cells leading to increased MAPK signaling and a more CAF-like phenotype (described in [77]). Interestingly, miR-211 has also been detected in melanoma sEVs and was shown to be induced upon treatment with the BRAF inhibitors vemurafenib and dabrafenib. It was proposed that expression is induced through MITF and leads to increased proliferation of parent melanoma cells [78]. To date, there is limited knowledge about recruitment of RNAs into melanosomes and sEVs. Whether the overlapping presence of MITF-regulated miR-211 in sEVs and melanosomes suggests a crosstalk of cargo-loading into these compartments remains to be resolved.

## 4. The Role of EVs in Melanoma Tumor Niche Formation

“When a plant goes to seed, its seeds are carried in all directions; but they can only live and grow if they fall on congenial soil”. Already in 1889 Stephen Paget applied the famous “seed-and-soil” concept to the dissemination of cancer cells and recognized the importance of the microenvironment on tumor spread. Indeed, primary tumors manipulate healthy tissue to facilitate metastasis formation, a process that has been coined as establishing the premetastatic niche (PMN) [79]. PMN formation encompasses several cell types and processes including increase in vascular permeability [80], recruitment of bone-marrow derived cells (BMDCs), activation of CAFs [81], remodeling of the extracellular matrix [82], interaction with adipocytes [83] and suppression of immune regulators (reviewed in [84,85]). Secretion of proteins, including the heparan sulfate binding growth factor midkine (MDK), is crucial for PMN formation in melanoma [86] (reviewed in [87]). Moreover, as mediator of cell-to-cell communication, sEVs are important players in preparing PNM, which has recently been reviewed across cancers in general [88]. Consequently, sEVs have also been shown to influence metastatic organotropism i.e., the preferential spread of tumor cells to certain organs [22,89]. In a seminal study, Hoshino and colleagues showed that sEV integrins can direct metastatic spread to specific organs based on sEV-cell tropism: while a combination of α6β4/α6β1 sEV integrins was associated with lung metastases, expression of αvβ5 sEV integrins was linked to liver metastases. The authors proposed a model whereby integrins on the surface of sEVs may provide the molecular address to mediate sEV interaction with cell-associated extracellular matrix of a specific organ and subsequent uptake into that organ [22].

### 4.1. Angiogenesis

**Figure 3:**
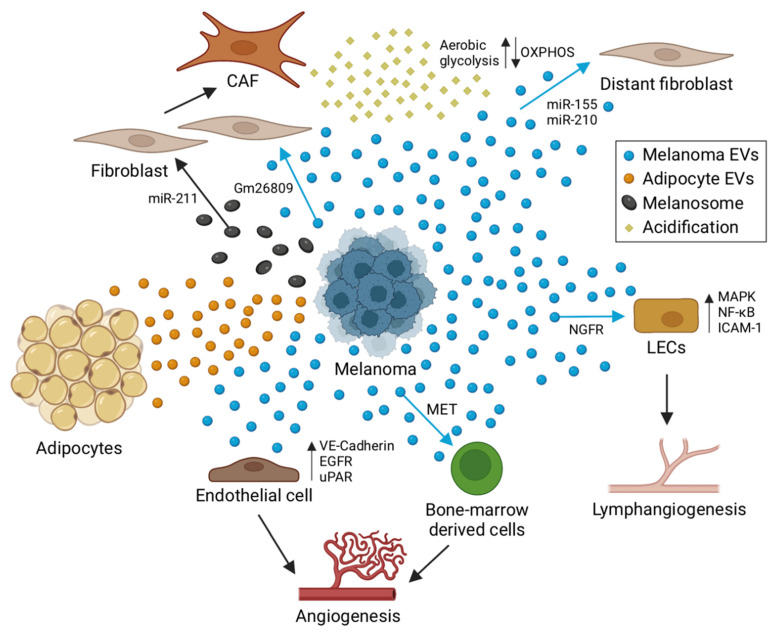
Role of EVs in melanoma tumor niche formation. CAF indicates cancer-associated fibroblast; OXPHOS refers to oxidative phosphorylation. Created with BioRender.com, accessed on 18 May 2022.

To cover the need for increased oxygen and nutrient supply, metastasizing cancer cells induce the formation of blood vessels (angiogenesis), the remodeling of pre-existing vasculature, and the recruitment BMDCs, which stimulate these processes. A role for melanoma-derived sEVs in angiogenesis and activation of bone-marrow-derived cells has been addressed in several studies. For example, sEVs have been shown to steer bone-marrow-derived cells towards a pro-vasculatory phenotype and to promote the formation of the lung pre-metastatic niche by horizontal transfer of the sEV-derived MET oncoprotein [90] (Figure 3). Similarly, in a replication study, sEV education in the same B16F10 mouse led to a slight increase in lung and femur metastases, which was reduced when administering sEVs with diminished MET expression, although the changes were not statistically significant [91]. Another study showed that melanoma sEVs expressing uPAR can promote angiogenesis by inducing VE-Cadherin, EGFR and uPAR and ERK1,2 signaling in endothelial cells [92].

### 4.2. Lymphangiogenesis

Besides traditional angiogenesis, melanoma can induce lymphangiogenesis (growth of lymphatic vessels), which has been associated with the incidence of sentinel lymph node metastasis and decreased disease-free survival [93]. Lymph node conditioning by sEVs was first described by Hood and colleagues in 2011. They reported an impact of melanoma sEVs on lymph node organization and recruitment of melanoma cells to exosome-rich sites in sentinel lymph nodes [94]. In a more recent study, García-Silva and colleagues showed that sEVs can stimulate lymphangiogenesis and metastasis through NGFR-mediated signaling [25]. Consistently, ablation and inhibition of NGFR in melanoma sEVs diminished lymph node metastasis and increased survival in a B16 mouse model. They also reported an induction of MAPK, NF-κB, and ICAM-1 signaling in lymphatic endothelial cells (LECs) by melanoma sEVs. An interaction of melanoma sEVs with LECs was confirmed in another study by Leary and colleagues. They showed that sEVs are transported and taken up by LECs in a VCAM-1 dependent fashion, which led to gene expression changes and proliferation in these cells. Additionally, sEVs impaired tumor immunity by shuttling MHC-1 to LECs [95].

### 4.3. Tumor–Stroma Interactions

Further, melanoma-derived sEVs have been implicated in regulating the transformation of fibroblasts into cancer-associated fibroblasts. For example, mouse melanoma sEVs from B16F0 cells were able to program NIH/3T3 towards increased CAF marker (α-SMA and FAP) expression and cell migration. In turn, sEV-conditioned NIH/3T3 cells induced melanoma cell (Cloudman S91) proliferation and migration in a co-culture system. This effect partially depended on the long non-coding RNA Gm26809, which was highly expressed in melanoma sEVs. Thus, the authors proposed a model whereby melanoma sEV-derived Gm26809 is delivered to target fibroblast cells to reprogram them into CAFs [96]. A further study showed that melanoma-derived sEVs can induce tumor-promoting proinflammatory activity of CAFs [97]. Interaction of cancer cells and CAFs can also affect cell metabolism. The concept of a “reverse Warburg effect” has been proposed, whereby the stroma may contribute to tumor progression by reprogramming their metabolism to aerobic glycolysis and providing invading cancer cells with the resulting metabolites e.g., lactate and pyruvate [98]. sEVs may be involved in inducing this reverse Warburg effect in CAFs, as melanoma sEV-derived miR-155 and miR-210 were shown to be required for enhancing aerobic glycolysis and reducing oxidative phosphorylation (OXPHOS) in adult human fibroblast cells, a metabolomic change that was associated with extracellular acidification [99]. Exosomal miR-155 has also been shown to induce a proangiogenic switch by directly targeting the suppressor of cytokine signaling 1 (SOCS1) which leads to JAK2/STAT3 activation and consequently increased expression of VEGFa, FGF2, and MMP9 in fibroblasts [100].

sEV regulation in the reverse direction from stroma to the tumor has also been observed. Lazar and colleagues showed that adipocyte-derived sEVs lead to increased migration and invasion [101]. In addition, they showed that adipocyte sEVs contain proteins linked to fatty acid oxidation (FAO). Interestingly FAO in melanoma was increased in melanoma cells in the presence of adipocyte sEVs, potentially explaining the negative impact of obesity on melanoma prognosis [101]. A more recent study demonstrated direct transfer of the FAO protein machinery and FA substrates to melanoma cells through sEVs [102] and thus confirming the previous connection between adipocyte sEVs and melanoma metabolism. Given these promising in vitro results it will be interesting to test adipocyte sEV–melanoma interactions in vivo.

## 5. Profiling of EV Cargo for Potential Biomarker and Drug Target Discovery

In a simplified view, one can consider EVs as biological containers that either maintain cellular homeostasis (by degrading, recycling or storing molecular cargo) or communicate to the surrounding tissues (by delivering molecular cargo). Since the EV cargo is specific to a certain cell type, EVs can provide a molecular fingerprint of the cell of origin. Thus, EVs secreted from melanoma cells can serve as potential biomarkers for diagnosis and/or progression. Moreover, as EVs contain several different types of molecules including mRNAs, miRNAs, long non-coding RNAs, mitochondrial DNAs, single-stranded DNAs, double-stranded DNAs, proteins, metabolites and lipids [26], analysis of EVs may provide a set of biomarkers that can be used in combination. In recent years, attempts have been made to profile the molecular content of EVs from cell culture supernatant and body fluids, whereby blood plasma and serum are the most used sources.

### 5.1. EV-Derived miRNA Profiling in Melanoma Cells

Due to their promiscuous nature, EV-derived miRNAs can have a broad impact on signaling in parent and recipient cells and are therefore of particular interest for biomarker studies. To date, most insights on melanoma sEV-derived miRNA have been obtained through microarray analysis. With better genomic/transcriptomic technologies being developed, more in-depth sEV profiling by RNA-sequencing (RNA-seq) will become feasible and a few isolated RNA-seq studies of melanoma sEVs have recently emerged.

In 2012, Xiao and colleagues performed microarray analysis of sEV-derived miRNAs of melanoma (A375) and melanocyte (HEMa-LP) cell lines. They found 228 differentially expressed miRNAs in melanoma sEVs compared to melanocyte sEVs (Table 1) [103]. A total of 70 of these miRNAs were associated with cancer based on ingenuity pathway analysis and a further 15 miRNAs (let-7c, miR-138, miR-125b, miR130a, miR-34a, miR-196a, miR-199b-3p, miR-25, miR-27a, miR-200b, miR-23b, miR-146a, miR-613, miR-205, miR-149) were associated with melanoma metastasis [103]. Among the differentially regulated miRNAs, miR-31 and miR-185 were known tumor suppressors in melanoma [104,105] and hsa-miR-34b was shown to target the oncogene MET, which is associated with melanoma invasiveness [106]. Furthermore, for many of the other identified sEV miRNAs (e.g., let-7a, miR-182, miR-221, miR-222, miR-31, miR-19b-2, miR-20b and miR-92a-2, miR-21, miR-15b, miR-210, miR-30b, miR-30d, and miR-532-5p) a cellular function important in melanoma has been described and recently been reviewed [107,108]. In particular, the miR-221/222 pair plays a prominent role in the progression of several cancers (reviewed in [109]). In melanoma, miR-211/222 have been associated with activation of oncogenic pathways through deregulation of p27 and c-KIT [110,111], making the miRNAs interesting clinical targets. Notably, Felicetti and colleagues found that sEV-derived miR-222 (but not miR221) can increase malignancy in patient-derived melanoma cells involving the PI3K pathway [112].

An additional study analyzed sEV-derived miRNAs after immunomagnetic enrichment. Rappa and colleagues profiled FEMX-1 melanoma sEVs that were enriched for the pentaspan transmembrane protein prominin-1/CD133. Prominin-1 had been previously shown to be expressed in high levels on sEVs from metastatic cells [126]. Microarray analysis revealed 49 differentially expressed miRNAs in sEVs compared to parent cells, of which 13 were associated with cancer and metastasis [113]. Promising upregulation was detected for miR-216b, let-7i and miR-10a. Notably, miR-10 family members are highly conserved miRNAs within hox clusters [127] and possess important functions in development and cancer, which have been reviewed in [128]. Similarly, let-7 family miRNA members are conserved across species [13] and have important functions in stem cell maintenance and epithelial to mesenchymal transition [129]. Specifically, melanoma-EV derived let-7i was found to be associated with phenotype switching of melanocytes through MAPK signaling [121]. To conclude, sEV isolation, based on stem-cell marker expression such as prominin-1/CD133, may be used for selection of EV-derived miRNAs associated with malignancy.

Furthermore, miRNA profiling of sEVs has been carried out in mouse models. For example, Bland and colleagues studied miRNAs and mRNAs of mouse melanoma B16F0-derived sEVs and parent cells by an Affymetrix microarray [114]. Of the 293 miRNAs detected above background, 30 were exclusive to sEVs and 139 were specific to parent cells, whereas the rest were present in both compartments. Among the highly enriched sEV-derived miRNAs, they detected let-7 family members let-7c, let-7b, let-7d and let-7a [114]. An additional study performed small RNA sequencing of melanoma-derived sEV from B16F1 cells and found 168 miRNA, which covered 93.5% of EV miRNAs listed in ExoCarta [117].

Hypoxia is a common feature in solid tumors and is linked to cancer progression. Thus, the effect of hypoxic signaling on sEV miRNA cargo was addressed in four patient derived melanoma cells lines (DMBC9, DMBC10, DMBC11 and DMBC12) [115]. Microarray analysis revealed 298 shared miRNAs in normoxic and hypoxic sEVs, whereas 50 and 130 miRNAs were unique to normoxic and hypoxic sEVs, respectively. Three miRNAs (miR-125b-5p, miR-21-5p and miR-3934-5p) were higher in sEVs under normoxic conditions, whereas 15 miRNAs were significantly higher in hypoxic sEVs [115].

miRNA profiling was additionally extended to different vesicle subtypes. In 2015, Lunavat and colleagues profiled EVs (exosomes and MVs) and apoptotic bodies of A375, MML-1 and SK-MEL-28 melanoma cells by deep-sequencing and detected distinct small RNA profiles and enrichment of sEV-derived miR-214-3p, miR-199a-3p and miR-155-5p, which are all linked to melanoma progression [116]. Importantly, analysis of clinical tissues revealed that multiple sEV miRNAs were specifically present in melanoma tissues, but not in benign naevi. In a follow-up study, Lunavat and colleagues sequenced EVs (exosomes and MVs) and apoptotic bodies of A375 and MML-1 cells upon vemurafenib treatment and found up-regulation of cellular and sEV-derived miR-211–5p under these conditions [78].

In another study Gerloff and colleagues compared melanoma cell line derived (WM9, WM35, and WM902B) and melanocyte-derived (NHEM) exosomes by next-generation sequencing and found 12 significantly enriched and 22 significantly decreased miRNAs. Among the top five candidates were miR-100-5p, miR-99b-5p, miR-221-3p, miR-24-3p, and miR-125b-5p [118]. miR-125b-5p was followed up further and was shown to be delivered to tumor-associated macrophages (TAM) by melanoma exosomes to partially induce a tumor-promoting phenotype [118]. In contrast to a tumor-promoting role of miR-125-5p, another study found that lower levels of serum-derived exosomal miR-125b are associated with advanced melanoma [119].

### 5.2. EV-Derived miRNA Profiling of Melanoma Liquid Biopsies

Due to the limited amount of material, RNA profiling of plasma- and serum-derived EVs remains challenging, but a handful of studies have performed microarray analysis and RNA-seq of sEVs isolated from liquid biopsies of melanoma patients.

A NanoString miRNA array was applied to plasma-derived sEV samples of a cohort of 36 patients including sporadic metastatic melanoma patients, unaffected, and affected familial melanoma (CDKN2A/p16 gene alteration carriers) patients and healthy controls. Notably, sEV miRNAs miR-17, miR-19a, miR-21, miR-126, and miR-149 showed increased levels in metastatic sporadic melanoma patients compared with the other groups. There was no significant difference between familial melanoma patients and unaffected healthy control samples. This may suggest that miRNAs play a less prominent role in the onset of familial melanoma and that sEV biomarkers may be able to distinguish between melanoma of different origins [120]. Of note, in another study, EMT regulators miR-191 and let-7a were significantly higher in serum-derived sEVs of stage I melanoma patients compared to control patients in a cohort of 41 patients (20 nonmelanoma vs. 21 stage I melanoma) [121]. In a more recent study, RNA-seq of plasma-derived sEVs from a patient cohort of 20 patients per group (melanoma vs. healthy control patients) and a validation cohort of 28 patients per group revealed miR-1180-3p as a novel potential diagnostic marker for cutaneous melanoma [122] with an area under the curve (AUC) value of 0.729. In functional experiments, miR-1180-3p reduced proliferation, migration, and invasion of melanoma cells and was negatively regulated by ST3GAL4 [122]. A further study revealed a potential homeostatic role of sEVs in regulating miR-494. Li and colleagues reported miR-494 to be enriched in sEVs of melanoma cell lines and melanoma serum compared to parent cells and healthy control serum, respectively [123]. By blocking exosome release with Rab27 and overexpressing miR-494 they claimed that the resulting intracellular accumulation of miR-494 prevented melanoma growth and metastasis [123]. Potentially promising EV-derived miRNA biomarkers for a clinical setting were obtained in a study by Tengda and colleagues [124]. They reported that EV-derived miRNA-532-5p and miRNA-106b separate healthy melanoma patients from healthy donor serum samples with AUCs of 0.867 and 0.820, respectively, and an AUC of 0.936 in combination. In a blinded test of 25 melanoma and 25 healthy patients, a panel combining miR-532-5p and miR-106b identified melanoma patients with a sensitivity of 92% and a specificity of 88% [124].

sEV microarray analyses have also been carried out in uveal melanoma, a distinct type of melanoma that occurs within the eye. Uveal melanoma patients revealed higher levels of miR-146a in serum and serum-derived sEVs compared to control patients. Notably, higher miR-146a levels in formalin-fixed, paraffin-embedded samples potentially pointed to a tumor autonomous function of miR-146a. Thus, miR-146a might be a potential circulating biomarker for uveal melanoma [125]. Since biological pathways of uveal melanoma differ substantially from cutaneous melanoma, it remains to be explored whether this finding can be translated to cutaneous melanoma.

### 5.3. EV-Derived Protein Profiling in Melanoma Cells

Proteomic analysis of EVs is still at an early phase and standardized methods will need to be established to isolate biomarkers that are consistent across different studies. In 2004, Mears and colleagues were the first to perform proteomic analysis of melanoma sEVs. They compared sEVs and cell lysates of SK-MEL28 and MeWo cells. The study reported sEV enrichment of proteins including p120-catenin, radixin, and immunoglobulin superfamily member 8 IGSF8 (PGRL). They also observed a general reduction of lysosmal and mitochondrial proteins in sEVs in contrast to cell lysates [130].

The study of Xiao and colleagues mentioned in Section 5.1 [103] also analyzed proteomic profiles of melanocytic (HEMa-LP) and melanoma (A375) sEVs and found differentially expressed proteins including upregulated proteins annexin A1, annexin A2, syntenin-1, and hyaluronan and proteoglycan link protein 1 (HAPLN1), which have functions related to angiogenesis, melanoma cell invasion, migration, and metastasis [131,132,133,134]. OXCT and MFGE8 are also worth mentioning, since OXCT has recently been identified as a rate-limiting enzyme for ketone metabolism in cancer [135] and MFG-E8 reportedly enhanced melanoma tumorigenicity through Akt- and Twist-dependent pathways [136]. Of note, annexin A2 was also detected in another study that profiled proteins and miRNAs in prominin-1/CD133-enriched sEVs. Besides Annexin A2, prominin-1/CD133-enriched sEVs comprised 154 other proteins including pro-metastatic proteins CD44, MAPK4K, GTP-binding proteins, and ADAM10 [113]. Furthermore, an additional study by Lazar and colleagues evaluated sEV proteomes of different melanoma cell lines that were classified as nontumorigenic (MNT-1, G1, 501mel), tumorigenic (SKMEL28 and Daju), and metastatic (A375M and 1205Lu) based on a xenograft model. They found that metastatic sEVs differed most and showed a specific signature [137]. Interestingly, exposure of less metastatic cell lines to sEVs of metastatic cell lines increased their migration [130]. In addition, an in vitro study by Guerreiro and colleagues compared sEVs of oral squamous cell carcinoma (E10), pancreatic ductal adenocarcinoma (BxPC3) and melanoma brain metastasis (H3) cell lines and found 25% of overlapping proteins [138]. All of the cancer sEVs showed the presence of proteins linked to tumorigenic processes (angiogenesis, inflammation, cell proliferation, migration, immunity, and cell adhesion) [138].

In another approach, the effect of pH modulation on sEV protein content was explored to model melanoma progression under microenvironmental acidic conditions. Analysis of Mel-501-derived sEVs profiled at normal conditions and at pH 6.0 revealed enrichment of proteins in acidic sEVs including HRAS, GANAB, CFL2, HSP90B1, HSP90AB1, GSN, HSPA1L, NRAS, HSPA5, TIMP3, and HYOU1, which correlated with poor prognosis. Notably, pH melanoma cells under normal pH gained migratory and invasive properties upon conditioning with sEVs obtained from acidic medium [139].

Whole proteome analysis has also been carried out in the B16 melanoma mouse model. B16F1 mouse melanoma-derived sEVs revealed proteins associated with molecular processes such as “Cell Death and Survival,” “Cellular Movement,” “Cell-to-Cell Signaling and Interaction,” “Cellular Growth and Proliferation,” and “Cell Morphology”, and most of the proteins overlapped with entries in ExoCarta (86.3%) [117]. Further proteomic profiling studies in the B16 melanoma mouse model are reviewed in [140].

### 5.4. EV-Derived Protein Profiling in Liquid Biopsies

To date, proteomic profiling of plasma and serum samples remains a challenge since high-abundant proteins (mostly albumin) dominate. In addition, plasma and serum contain a mixture of sEVs released from several cell types. Thus, most of current melanoma studies have either performed targeted analysis of blood sEV samples or aimed for increased sensitivity by enrichment of melanoma-specific sEVs. An early study focused on targeted analysis of proteins known to be linked to melanoma progression and found “melanoma inhibitory activity” (MIA) and S100B to be significantly enriched in sEVs of melanoma patients compared to healthy controls and disease-free patients, whereas TYRP2 showed no difference [141]. Another targeted approach designed an ELISA method to detect and capture sEVs, based on expression of the sEV marker CD63 and the tumor-associated marker caveolin-1. Notably, plasma-derived sEVs expressing CD63 or caveolin-1 were significantly increased in melanoma patients as compared to healthy controls [142].

In a seminal study, Peinado and colleagues performed proteomic profiling of metastatic mouse (B16-F10) and human (SK-Mel28/-202/-265/-35) melanoma cells to identify a potential diagnostic signature in melanoma-derived sEVs. Subsequent analysis of selected proteins in sEVs of stage I, stage III, stage IV melanoma patients and healthy controls revealed a potential diagnostic “signature”, which included the melanoma-specific protein TYRP2, VLA-4, HSP70, an isoform of HSP90 and oncoprotein MET [90].

To enrich for melanoma-specific sEVs, Pietrowska and colleagues used immunoselection to capture CSPG4/MCSP-positive sEVs from melanoma patients [143]. CSPG4/MCSP had previously been identified as a marker of melanoma cells [144,145] and was used in a preceding study to establish an immunoaffinity-based method to isolate melanoma-specific sEVs, which were characterized by enrichment of melanoma-associated antigens (MAAs), such as TYRP2, MelanA, GP100, and VLA4 compared to non-captured sEV [146]. Proteomic profiling of melanoma-specific sEVs (CSPG4-captured) and non-malignant sEVs (non-captured) of 15 melanoma patients revealed 73 overexpressed proteins, of which 16 could discriminate melanoma-specific from non-malignant sEVs. Notably, the protein profiles of melanoma-specific sEVs could also stratify melanoma patients into patients with no evidence of melanoma after therapy vs. patients with progressive disease. PDCD6IP (ALIX), which was highly expressed in melanoma-specific sEVs of progressing patients, had the strongest discriminating power. In contrast, CNTN1 (contactin-1) was upregulated only in melanoma-specific sEVs of patients that did not progress [143]. In a recent study García-Silva and colleagues isolated and profiled sEVs from the fluid that was collected from the lymphatic drainage (exudative seroma) of stage III melanoma patients and showed that seroma-derived EV proteins resemble melanoma progression [147]. Thus, exudative seroma might be a valuable alternative EV source to plasma and serum for biomarker discovery.

### 5.5. Profiling of Less Studied EV Cargo

Although sEV-derived miRNA and proteins have received the most attention, other sEV cargos are increasingly gaining interest. For example, in a recent study Shi and colleagues profiled sEV-derived mRNAs in melanoma patients’ plasma to find biomarkers for predicting and monitoring the response to immune checkpoint blockade inhibitors [148]. This complemented earlier studies, which analyzed sEV-derived mRNAs in melanoma cells, and have recently been reviewed in the context of several cancers [149]. Furthermore, the therapeutic potential of sEV-derived long noncoding RNA in cancer has recently been reviewed [150]. In contrast to circulating tumor DNA (ctDNA) that is released into the blood by dying cells, EV-derived ctDNA originates from living cells and may therefore provide complementary information to ctDNA analysis. Although several studies have analyzed ctDNA in plasma of melanoma patients, only one assessed EV-derived ctDNA to date. In that study, Zocco and colleagues detected EV-derived ctDNA in the plasma of metastatic melanoma patients, which improved the detection of BRAF mutant DNA in these patients [151]. Furthermore, lipid profiles of melanoma sEVs have been explored. Lipidomics of sEVs derived from LCP and SK-Mel28, which are distinguished by different metastatic behavior, have shown more saturated and shorter fatty acid tails in poorly metastatic (LCP) cells compared with highly metastatic (SK-Mel28) cells [152]. The same study detected a lipid sEV marker, a peculiar phospholipid bis(monoacylglycero)phosphate [152]. To exclude cell-line-specific changes between unmatched SK-Mel28 and LCP cells, a comparative analysis of matched cell line pairs (e.g., A375 vs. A375M or WM793 vs. 1205Lu) would have been informative. A metabolomics study of cancer stem cells and melanoma serum has also been performed [153]. The study revealed pronounced differences between metabolomic profiles of healthy and melanoma patients. Due to the moderate sample size, there was not enough statistical power to define prognostic biomarkers, but based on these promising findings, further larger-scale metabolomic analysis will have great potential for isolation of biomarkers to predict melanoma progression.

## 6. Conclusions

To summarize, EVs are important modulators of melanoma progression and can regulate oncogenic processes at the level of tumor homeostasis as well as interaction with the tumor microenvironment. Numerous studies have revealed that the cross-talk of melanoma EVs with surrounding tissues can shape the metastatic process at several different levels, including angiogenesis, tumor–stroma interactions, and lymphangiogenesis. In addition, interactions of EVs with the immune system have a great impact on melanoma biology. Immunological aspects have been reviewed in recent studies [154,155] and were not addressed here. Moreover, several components of EV cargo have reportedly been linked to melanoma malignancy, and emerging profiling studies of plasma- and serum-derived EVs have identified potential biomarkers for melanoma onset and metastasis formation. Given the diversity of the cargo in a particular EV, multiplexed analysis of different molecules such as RNAs, proteins, and metabolites will allow for the identification of combined EV signatures for improved diagnostics. The large heterogeneity within EV populations still poses a difficult challenge for EV studies. Thus, there is a need for new technologies for EV isolation and characterization coupled with improved EV-omics analysis to further lift the potential of EVs in diagnosis, monitoring, and treatment of melanoma and other cancers.

## Figures and Tables

**Table 1:** Studies detecting sEV-derived miRNA implicated in melanoma biology.

miRNAs	sEV Source	sEV Marker	sEV Isolation	RNA Isolation Kit/Quantitation Method	Study
miR-31, miR-185, miR-34b	HEMa-LP, NHEM-c, A375, SK-MEL-28	CD81+, HSC70+ Calnexin-, cytochrome c-	UF ^1^ + UC ^2^	mirVana/Microarray	[103]
miR-222	PD cell lines	HSP90 TSG101 LAMP2 CD63 RAB5B	UC or EQ ^3^	NorgenBioteK/qRT-PCR ^4^	[112]
miR-216b, let-7i, miR-10a	FEMX-I	Alix	ImaSep ^5^ + UC	Qiazol/Microarray	[113]
let-7c, let-7b, let-7d, let-7a	B16F0	Hsp70+,CD63+ CD9+ CD81-	UC	RNAeasy, Trizol/Microarray	[114]
miR-494-5p, miR-4497, miR-513a-5p (high in hypoxic sEVs) vs. miR-125b-5p, miR-21-5p, miR-3934-5p (high in normoxic sEVs)	DMBC9, -10, -11, -12	CD9+, CD63+	UC	miRCURY/Microarray	[115]
miR-214-3p, miR-199a-3p, miR-155-5p	A375, MML-1, SK-MEL-28	Exo ^6^: FLOT1+, TSG101+ MV^7^: FLOT1+, TSG101 APB ^8^: FLOT1+, BCL2+, Calnexin+, TSG101	UC	miRCURY/Ion Torrent	[116]
miR-211	MML-1, A375	Exo: CD81+, TSG-101+, CD81- MV: absence of TSG-101- APB: Calnexin+	UC	miRCURY/Ion Torrent	[78]
168 miRNAs	B16F1	CD9+, CD63+ CD81+, HSP70+	UC	Zymo Research/SOLiD 5500	[117]
miR-100-5p, miR-99b-5p, miR-221-3p, miR-24-3p, miR-125b-5p,	WM9, WM35, WM902B, NHEM	CD63+, CD81+ Calnexin-	UC	TriFast™/Illumina	[118]
miR-125b, miR-16	Plasma	-	EQ	TRIzol/ qRT PCR	[119]
miR-17, miR-19a, miR-21, miR-126, miR-149	Plasma	-	EQ	Qiazol + Qiagen miRNeasy/ NanoString	[120]
miR-191 and let-7a	HEMa-LP, NHEM, A375 SK-MEL-28 Serum	-	UC+EQ	mirVana, SeraMir, qRT-PCR	[121]
miR-1180-3p	ME4405, A375, SK-MEL-5, SK-MEL-28, plasma	-	UC	-	[122]
miR-494	A375, serum	-	UC	TRIzol/qRT-PCR	[123]
miRNA-532-5p, miRNA-106b	serum	CD63+	UC	Sangon Biotech/qRT-PCR	[124]
miR-146a ^11^	VH ^9^, serum, FFPE ^10^	CD9+/−, CD63++, CD81+	UC	Qiagen miRNeasy/Microarray	[125]

^1^ UF, ultrafiltration; ^2^ UC, ultracentrifugation; ^3^ EQ, ExoQuick; ^4^ qRT-PCR, quantitative real-time polymerase chain reaction; ^5^ ImaSep, immune magnetic separation; ^6^ Exo, exosome; ^7^ MV, microvesicle; ^8^ APB, apoptotic body; ^9^ VH, vitreous humor; ^10^ FFPE, formalin-fixed/paraffin-embedded; ^11^ miR-146a, measured in uveal melanoma samples.
